# Region-independent active CNS net uptake of marketed H^+^/OC antiporter system substrates

**DOI:** 10.3389/fncel.2024.1493644

**Published:** 2024-10-29

**Authors:** Frida Bällgren, Yang Hu, Shannuo Li, Lara van de Beek, Margareta Hammarlund-Udenaes, Irena Loryan

**Affiliations:** Department of Pharmacy, Faculty of Pharmacy, Translational Pharmacokinetics-Pharmacodynamics Group, Translational Pharmacokinetics Pharmacodynamics (tPKPD), Uppsala University, Uppsala, Sweden

**Keywords:** pyrilamine-sensitive proton-coupled organic cation (H^+^/OC) antiporter, neurotoxicity, active uptake, blood-brain barrier, blood-spinal cord barrier, blood-CSF barrier, K_p,uu_, pH modulation

## Abstract

The pyrilamine-sensitive proton-coupled organic cation (H^+^/OC) antiporter system facilitates the active net uptake of several marketed organic cationic drugs across the blood-brain barrier (BBB). This rare phenomenon has garnered interest in the H^+^/OC antiporter system as a potential target for CNS drug delivery. However, analysis of pharmacovigilance data has uncovered a significant association between substrates of the H^+^/OC antiporter and neurotoxicity, particularly drug-induced seizures (DIS) and mood- and cognitive-related adverse events (MCAEs). This preclinical study aimed to elucidate the CNS regional disposition of H^+^/OC antiporter substrates at therapeutically relevant plasma concentrations to uncover potential pharmacokinetic mechanisms underlying DIS and MCAEs. Here, we investigated the neuropharmacokinetics of pyrilamine, diphenhydramine, bupropion, tramadol, oxycodone, and memantine. Using the Combinatory Mapping Approach for Regions of Interest (CMA-ROI), we characterized the transport of unbound drugs across the BBB in specific CNS regions, as well as the blood-spinal cord barrier (BSCB) and the blood-cerebrospinal fluid barrier (BCSFB). Our findings demonstrated active net uptake across the BBB and BSCB, with unbound ROI-to-plasma concentration ratio, K_p,uu,ROI_, values consistently exceeding unity in all assessed regions. Despite minor regional differences, no significant distinctions were found when comparing the whole brain to investigated regions of interest, indicating region-independent active transport. Furthermore, we observed intracellular accumulation via lysosomal trapping for all studied drugs. These results provide new insights into the CNS regional neuropharmacokinetics of these drugs, suggesting that while the brain uptake is region-independent, the active transport mechanism enables high extracellular and intracellular drug concentrations, potentially contributing to neurotoxicity. This finding emphasizes the necessity of thorough neuropharmacokinetic evaluation and neurotoxicity profiling in the development of drugs that utilize this transport pathway.

## 1 Introduction

Despite significant progress in recent years, the development of drugs for treating central nervous system (CNS) diseases continues to face higher attrition rates compared to non-CNS drugs (Aesselheim et al., 2015; Authier et al., 2016). This is often due to the lack of efficacy of treatment or problems with safety profiles. One major challenge in this field is drug-induced CNS neurotoxicity, adverse events triggered by the malfunction of CNS organs due to drug use (Edwards and Aronson, 2000). Such neurotoxicity is a significant concern in clinical practice and during drug development, frequently leading to substantial attrition rates, especially in the later stages of clinical development (Cook et al., 2014). Currently, predicting drug-induced neurotoxicity during preclinical assessments remains challenging, leaving a critical gap in the creation of innovative and safe medications. There is still an incomplete understanding of the complex pathways and mechanisms that lead to central drug-induced neurotoxicity, particularly in the cases of drug-induced seizures (DIS) and mood- and cognitive-related adverse events (MCAEs).

Existing pharmacovigilance databases offer a unique opportunity to explore drug-induced neurotoxicity using novel approaches. The FDA Adverse Event Reporting System (FAERS) database, for instance, provides a rich source of adverse event data submitted voluntarily by drug manufacturers, healthcare professionals, and consumers. Within the framework of the EU IMI2-NeuroDeRisk consortium, mining the FAERS database followed by disproportionality analysis and expert curation has identified marketed drugs with a high occurrence of DIS and MCAEs (Andronis et al., 2020; Lipponen et al., 2023). Notably, many of the top-ten drugs with the highest occurrence in each category were organic cations linked to the pyrilamine-sensitive putative proton-coupled organic cation (H^+^/OC) antiporter system and some organic cation transporters (OCTs) (Doetsch et al., 2022). Among the drugs associated with DIS were diphenhydramine, bupropion, tramadol, amitriptyline, clozapine, and venlafaxine (Lipponen et al., 2023). Meanwhile, drugs associated with MCAEs included memantine, varenicline, and metoclopramide (Andronis et al., 2020). These findings align with various reports documenting DIS and MCAEs for many of the listed drugs, including diphenhydramine (Kim et al., 2023; Köppel et al., 1987; Alldredge et al., 1989; Thundiyil et al., 2007; Jang et al., 2010), bupropion (Thundiyil et al., 2007; Finkelstein et al., 2013), tramadol (Thundiyil et al., 2007; Babahajian et al., 2019), and memantine (Witter et al., 2015; Moellentin et al., 2008). Interestingly, other substrates of the H^+^/OC antiporter system including pyrilamine (Kim et al., 2023; Kamei et al., 2000) and oxycodone (Klein et al., 2005) have also been associated with DIS.

Substrates of the H^+^/OC antiporter system, several of the identified drugs, including diphenhydramine, bupropion, tramadol, and memantine, exhibit unique properties reflected in their active net uptake across the blood-brain barrier (BBB) (Table 1). The active uptake of substances across the BBB was first documented in rats for oxycodone, using brain microdialysis (Boström et al., 2006). Microdialysis allows for the continuous measurement of the unbound (free) concentration of a drug in various compartments, including the brain and blood followed by the estimation of the unbound brain-to-plasma concentration ratio, K_p,uu,brain_ (Gupta et al., 2006). K_p,uu,brain_ is a key neuropharmacokinetic parameter that quantitatively characterizes the extent of unbound drug crossing the BBB, with a K_p,uu,brain_ above unity indicating predominant active influx (Hammarlund-Udenaes et al., 2008). According to the free drug theory, only unbound and unionized molecules can cross biological membranes. However, as organic cations, these drugs are predominantly ionized at physiological pH, necessitating a carrier-mediated transport system, such as the H^+^/OC antiporter, to facilitate their movement from the blood into the brain interstitial fluid (i.e., extracellular fluid). Further intra-brain distribution of these organic cations into cells is thought to primarily occur via passive diffusion, driven by pH partitioning mechanisms (Fridén et al., 2011).

**Table 1:** Summary of findings related to the association of investigated drugs with influx transporters and the reported K_p,uu_ values generated using cerebral microdialysis.

**Drug**	**Influx transporter/antiporter**	**Reported K_p,uu_**
Pyrilamine	Proton-coupled organic cation antiporter (Okura et al., 2008; Kitamura et al., 2016) SLC22A4, Organic Cation/Carnitine Transporter 1, OCTN1 and SLC22A5, Organic Cation/Carnitine Transporter 2, OCTN2 (Grigat et al., 2007; Ohashi et al., 2002; Yabuuchi et al., 1999) SLC35F2 (Mochizuki et al., 2021) SLC49A4, Disrupted In Renal Carcinoma 2, DIRC2 (Akino et al., 2023)	2.50 (Kitamura et al., 2016)
Diphenhydramine	Proton-coupled organic cation antiporter (Kitamura et al., 2016) Drug/H^+^-antiporter (Auvity et al., 2017) Amine/H^+^ antiporter (Sadiq et al., 2011) SLC22A1, Organic Cation Transporter 1, OCT1 (Boxberger et al., 2018) SLC35F2 (Mochizuki et al., 2021)	5.5 (Sadiq et al., 2011) 3.85 (Kitamura et al., 2016) 10.6 (Kawase et al., 2021a) 3.24 (Kawase et al., 2021b) 5.4-3.0 (Hu et al., 2018)
Bupropion	Proton-coupled organic cation antiporter (Doetsch et al., 2022)	1.9 (Cremers et al., 2016)
Tramadol	Proton-coupled organic cation antiporter (Kitamura et al., 2016, 2014)	2.90 (Kitamura et al., 2016) 2.30 (Kitamura et al., 2014)
Oxycodone	Proton-coupled organic cation antiporter (Okura et al., 2008; Kitamura et al., 2016) Amine/H^+^ antiporter (Sadiq et al., 2011) SLC35F2 (Mochizuki et al., 2021)	3.0 (Boström et al., 2006) 3.3 (Sadiq et al., 2011) 1.69 (Kitamura et al., 2016) 4.4 (Bällgren et al., 2023)
Memantine	Proton-coupled organic cation antiporter (Kawase et al., 2021a; Mehta et al., 2013; Higuchi et al., 2015) SLC22A2, Organic Cation Transporter 2, OCT2 (Busch et al., 1998)	1.80 (Kitamura et al., 2016) 2.03 (Kawase et al., 2021a)

DIS (ictogenicity), and MCAEs liability have been attributed to various pharmacodynamic mechanisms, such as deficit in inhibitory gamma-aminobutyric acid or an elevated glutamatergic excitatory signaling, or disbalance in central histaminergic signaling pathways, leading to impairment of normal neurological functions in various brain regions (Andronis et al., 2020; Lipponen et al., 2023; Larson et al., 2021; Hu et al., 2012). A common feature identified across different mechanisms is the brain region selectivity, which has often been associated with the expression level of pharmacodynamic targets. Variations in the extra- and intracellular concentrations of these substrates at specific target sites may play a critical role in the manifestation of neurotoxic effects. Despite this, there has been no systematic investigation into the brain regional distribution of H^+^/OC antiporter substrates associated with DIS or MCAEs.

The primary aim of this preclinical study was to deepen our understanding of the CNS regional disposition of H^+^/OC antiporter substrates at therapeutically relevant plasma concentrations. By exploring the neuropharmacokinetics of these compounds, particularly their passage across the brain and spinal cord barriers, we seek to uncover potential pharmacokinetic mechanisms underlying DIS and MCAEs. To achieve this, we employed the Combinatory Mapping Approach for Regions of Interest (CMA-ROI) methodology (Loryan et al., 2014, 2016), which allows for detailed characterization of the extent of BBB transport in specific CNS regions, including the frontal and parietal cortices, cerebellum, hippocampus, striatum, and the whole brain in rats. Additionally, we assessed the extent of transport across the blood-spinal cord barrier (BCSB) and the blood-cerebrospinal fluid barrier (BCSFB). The study also investigated the intra-brain distribution of selected drugs and their sensitivity to changes in brain intracellular pH. The selected compounds for this systematic investigation included pyrilamine, diphenhydramine, bupropion, tramadol, oxycodone and memantine. Some of the known transport characteristics for the selected drugs are presented in Table 1 (Auvity et al., 2017; Hu et al., 2018; Mehta et al., 2013; Higuchi et al., 2015; Kitamura et al., 2016; Kawase et al., 2021a,b; Mochizuki et al., 2021; Boxberger et al., 2018; Kitamura et al., 2014; Busch et al., 1998; Grigat et al., 2007; Percie du Sert et al., 2020; Bällgren et al., 2023; Ohashi et al., 2002; Yabuuchi et al., 1999). With this study, we aim to provide new insights into the CNS regional neuropharmacokinetics of these drugs and their implications for neurotoxicity.

## 2 Materials and methods

### 2.1 Chemicals

Pyrilamine maleate, diphenhydramine, diphenhydramine-D5 (Cerilliant), bupropion hydrochloride, bupropion-D9, tramadol hydrochloride, tramadol-^13^CD3, oxycodone hydrochloride, oxycodone-D6 (Cerilliant), memantine hydrochloride, 4-(2-hydroxyethyl)-1-piperazineethanesulfonic acid (HEPES), 2-hydroxypropyl-β-cyclodextrin (HPβCD), dimethyl sulfoxide (DMSO) were obtained from Sigma-Aldrich (Steinheim, Germany). Memantine-D6 hydrochloride was purchased from Bio-techne Ltd (Abingdon, UK). Oxycodone hydrochloride (OxyNorm^®^, Mundipharma, Cambridge, England) was purchased from Distansapoteket Stockholm (Apoteket AB, Stockholm, Sweden). Acetonitrile and formic acid were purchased from Merck (Darmstadt, Germany). Water used in all experiments was purified using a Milli-Q Academic system (Millipore, Bedford, MA, USA).

### 2.2 Animals

All experiments were performed on drug-naïve male Sprague-Dawley rats weighing 250–300 g (Taconic, Lille Skensved, Denmark). Experiments were performed in accordance with guidelines from the Swedish National Board for Laboratory Animals, and approved by the Animal Ethics Committee of Uppsala, Sweden (Ethical Approval Dnr 5.8.18-12230/2019). All rats were housed in groups at 20 to 22°C under a 12-h light/dark cycle with *ad libitum* access to food and water. Data were reported according to ARRIVE 2.0 guidelines (Percie du Sert et al., 2020).

The study was not blinded or randomized. In total 91 animals were used. For *in vivo* pharmacokinetic studies, sample size per drug, was six to seven. Given the desired probability level (α = 0.05), effect size (Cohen's d = 1.2), and statistical power level (0.8), the sample size per group required for a two-tailed *t*-test study was estimated to be minimally six (Krzywinski and Altman, 2013). *In vitro* studies on plasma protein and brain tissue binding as well as brain slice assay were performed on a minimum of three animals per group.

### 2.3 Assessment of the extent of drug transport across the CNS barriers

For the determination of the brain regional/spinal cord partitioning coefficient (K_p,ROI_) and the extent of unbound drug transport across the BBB, BSCB and BCSFB (K_p,uu,ROI_, K_p,uu,CSF_) in the CNS regions an *in vivo* pharmacokinetic study was performed using the CMA-ROI approach (Loryan et al., 2016). To minimize the use of animals, we opted for a single time point assessment for K_p,ROI_ at steady-state. To ensure the achievement of steady-state in blood and tissues, each drug was administered intravenously to awake, healthy male Sprague-Dawley rats (*n* = 6–7 per drug) using a combination of loading and maintenance doses through a catheter surgically implanted in the femoral vein one day before the experiments (Supplementary Table S1). The dosing regimens were chosen based on simulations of the plasma profiles of the drugs, targeting clinically relevant therapeutic concentrations, using the Berkeley Madonna software package (version 8.3.18 for Windows, Berkeley, CA, USA). The loading dose was given as a rapid infusion over 10 min (except for oxycodone which was given without loading dose) to quickly reach the targeted total plasma concentration. To maintain the achieved target plasma concentration, a maintenance dose was infused over 4 h. Blood samples (ca 200 μL) were collected at 0, 60 (not performed for memantine), 120, 180, and 240 min after the start of the infusion via a catheter in the femoral artery. At 4 h while the infusion was ongoing, the rat was anesthetized with 2.5% isoflurane (Abbot Scandinavia, Solna, Sweden) supplemented with oxygen, secured in a stereotaxic apparatus and CSF was sampled from the *cisterna magna* (not performed for memantine). Thereafter, ca 10 mL of blood was sampled from the heart using cardiac puncture. Blood samples were centrifuged at 10,000 rpm for 5 min, and plasma was collected. Following decapitation, the brain was removed; one half, referred to as the whole brain, was collected, while the other half was dissected into regions of interest, including the cerebellum, frontal cortex, parietal cortex, striatum, and hippocampus. Due to the common practice of using the whole brain in the evaluation of the BBB transport, we have included the whole brain and made a comparison to it between the regions to support or negate the assumption of homogenous BBB transport. The spinal cord was also sampled, except for the memantine study. Brain and spinal cord samples were weighed and along with the blood and CSF samples were stored at−80°C pending analysis. Prior to analysis, the brain regions/spinal cord were individually homogenized on ice in 1:4 (w:v) phosphate-buffered saline pH 7.4 using ultrasonic processor VCX-130 (Sonics, Chemical Instruments AB, Stockholm, Sweden) or 4-place mini beads homogenizer (VWR, Stockholm, Sweden).

### 2.4 Estimation of plasma protein and drug CNS tissue binding using equilibrium dialysis

Equilibrium dialysis was employed to determine the fraction of unbound drug in plasma (f_u,plasma_), homogenates of whole-brain (f_u,brain_) and the CNS regions of interest (f_u,ROI_) including the frontal cortex, parietal cortex, cerebellum, striatum, hippocampus, and spinal cord. The experiment was performed based on previously published protocols (Loryan et al., 2016; Kalvass and Maurer, 2002; Gustafsson et al., 2019). Briefly, a Teflon 96-well plate (model HTD96b, HTDialysis LLC, Gales Ferry, CT, USA) with a cellulose membrane with a 12–14 kDa molecular weight cutoff was used. The cellulose membrane was treated according to the recommendations from the manufacturer before starting the experiment (HTDialysis LLC, Gales Ferry, CT, USA). Fresh plasma (n=3 rats per drug) was obtained on the day of the experiment and the pH of the plasma was adjusted to 7.40 using H_3_PO_4_. Undiluted plasma was individually spiked to a final concentration of 500 nM for pyrilamine, diphenhydramine, bupropion, tramadol, or oxycodone, and 500 ng/mL (2,788 nM) for memantine. Whole brain and CNS regions of interest (*n* = 3–4 rats per drug) were homogenized in 1:9 (w:v) PBS pH 7.40. Diluted brain samples were individually spiked to a final concentration of 1,000 nM. Hundred μL of undiluted spiked plasma samples or homogenates were dialyzed against equal volumes of PBS, pH 7.40, for 6 h at 37°C at 200 rpm in an incubator with orbital shaking (MaxQ4450 Thermo Fisher Scientific, NinoLab, Sweden). One to three technical replicates were tested per biological replicate. Drug recovery and thermostability in plasma and brain were analyzed in each experiment by sampling spiked samples before and after the 6-h incubation. A spiking recovery (i.e., recovery of the spiking drug concentration) and thermostability within 100 ± 30 % was considered acceptable, and all results fell within this range. At the end of the 6-h incubation, 50 μL PBS sample from the receiver chambers was transferred into a Corning^®^ 0.33 mL round-bottom polypropylene 96-well plate (VWR, Stockholm, Sweden) containing 50 μL blank plasma or 1:9 (w:v) brain homogenate using a multichannel pipette. Immediately after this step, 50 μL spiked plasma or brain homogenate was taken from the donor chambers and added to the same 96-well plate containing 50 μL of blank PBS. The 96-well plate was sealed with aluminum film, vortexed and stored at −20°C pending bioanalysis. The fraction of unbound drug in plasma was assessed as a ratio between buffer and plasma concentrations. The unbound fraction of drug in the diluted (D) CNS tissue homogenate (f_u,hD_) was calculated as:


(1) $$\begin{array}{l} {\text{f}}_{\text{u},\text{hD}}=\frac{{\text{C}}_{\text{buffer}}}{{\text{C}}_{\text{homogenate}}} \end{array}$$

Where C_buffer_ and C_homogenate_ represent the concentration determined in the buffer (receiver side) and CNS tissue homogenate (donor side) samples, respectively. Due to the dilution required for preparation of homogenates, f_u,hD_ was corrected for the dilution factor (D, being 10 in this case) to obtain the actual f_u,ROI_, as described below.


(2) $${\text{f}}_{\text{u},\text{ROI}}=\;\frac{{\text{f}}_{\text{u},\text{hD}}}{{\text{D }+\text{ f}}_{\text{u},\text{hD}}{-\text{ D}}^{*}{\text{f}}_{\text{u},\text{hD}}\;}$$

A fraction of unbound drug close to 0 indicates a very high plasma protein or CNS tissue binding, while a value close to 1 indicates very low binding.

### 2.5 Evaluation of drug binding and uptake using the brain slice assay

The brain slice method was used to estimate the binding and uptake of drugs into the brain tissue (Fridén et al., 2009; Loryan et al., 2013). The uptake of drugs into the brain tissue was assessed by determining the unbound volume of drug distribution in the brain (V_u,brain_, mL/g brain). This is measured by relating the total drug concentration in the brain slice and the unbound drug concentration in the artificial extracellular fluid (aECF) at equilibrium. Drug-naïve rats (n=3 per drug) were anesthetized with inhalation anesthesia using 5% isoflurane on the day of the experiment and the isolated brain was immediately placed into blank ice-cold aECF. Six 300 μm thick brain slices were prepared from one brain. Brain slices were transferred into the beaker containing 15 mL of pre-oxygenated aECF spiked with one of the selected drugs at final concentrations of 100 nM (pyrilamine, diphenhydramine, bupropion, tramadol, oxycodone) or 200 nM (memantine). A 5 h incubation was performed at 37°C in an orbital shaker with a rotation speed of 45 rpm and constant oxygen flow of about 75–80 mL per minute through a glass frit. After the 5 h incubation, the virtually protein-free aECF buffer was sampled by aspiration of 200 μL and dispensed in an Eppendorf tube containing 200 μL of blank brain homogenate (1:4, w:v) to match the matrix of the brain slice samples. This blank homogenate was prepared in advance by weighing the brain from drug naïve rats and homogenizing on ice in 4 volumes (w:v) of aECF, initially mechanically for a maximum of 1 min using a Heidolph mechanical stirrer followed by ultrasonication for three cycles (5 s “on” and 5 s “off”) at an amplitude of 50 %. Subsequently, the brain slices were individually removed, dried on filter paper, and weighed in 1.5 mL Eppendorf tubes. The slices were individually homogenized in 9 volumes (w:v) of aECF with an ultrasonic processor. The samples of aECF at time zero were taken to investigate the recovery of the compounds from the experiment (%). The thermostability of the compounds at 37°C was assessed after the 5-h incubation period in a parallel aECF solution without the presence of brain slices. A spiking recovery and thermostability within 100 ± 30 % was considered acceptable. All samples were stored at −20°C pending bioanalysis.

To investigate the impact of pH modulation on intra-brain distribution, the pH modulators monensin and bafilomycin A1 were used. Monensin acts as a Na^+^/H^+^ ionophore and bafilomycin A1 is a macrolide that inhibits vacuolar-type H^+^-ATPase, a membrane-spanning proton pump that acidifies intracellular organelles (Aowicki and Huczyński, 2013; Gagliardi et al., 1999). Concentrations of 50 nM for monensin and 10 nM for bafilomycin A1 were selected (Fridén et al., 2011; Shacka et al., 2006). Brain slices (n=2 rats per drug and per pH modulator) were first preincubated for 30 min with the pH modulator before adding the experimental drugs pyrilamine, diphenhydramine, bupropion, tramadol or oxycodone at final concentration of 100 nM. The rest of the experiment was performed as described above.

Assuming that the concentration of the compounds in virtually protein-free aECF at equilibrium is equal to the interstitial fluid concentration in the brain slice, V_u,brain_ (mL/g brain) was estimated using Equation 3 as a ratio of the amount of compound in the brain slice (A_brain_, nmol/g brain) to the measured final aECF concentration, i.e., unbound concentration (C_buffer_, μmol/L).


(3) $$\begin{array}{l} {\text{V}}_{\text{u},\text{brain}}=\frac{{\text{A}}_{\text{brain}}-{\text{V}}_{\text{i}}\cdot {\text{C}}_{\text{buffer}}}{{\text{C}}_{\text{buffer}}\cdot \left(1-{\text{V}}_{\text{i}}\right)} \end{array}$$

Where V_i_ (mL/g brain) is the volume of the surrounding brain slices layer of aECF. A V_i_ of 0.133 mL/g brain was determined using ^14^C-sucrose in a dedicated experiment according to previously published protocols (Fridén et al., 2009; Hu et al., 2024).

### 2.6 Bioanalysis of drugs using LC-MS/MS

The bioanalysis of selected drugs and respective internal standards (IS) in all samples was performed using reversed-phase liquid chromatography followed by detection with a tandem mass spectrometer (LC-MS/MS). Quantitation was performed using multiple reaction monitoring (MRM) mode to monitor Parent → Product ion (m/z) transitions. Xevo TQ-S micro (Waters, UK) equipped with electrospray ionization and operating in positive ion mode and the UPLC system ACQUITY (Waters Corporation, Taunton, Massachusetts, USA) was used for quantification of pyrilamine, diphenhydramine, bupropion, tramadol and oxycodone. Quatro Ultima^TM^ PT triple quadrupole mass spectrometer (Micromass, Waters, Manchester, United Kingdom) and an HPLC system equipped with two LC-10ADvp pumps and a SIL-HT autosampler (Shimadzu, Kyoto, Japan) were used for the quantification of memantine according to previously published protocol (Gustafsson et al., 2019). Five types of matrices were used in the preparation of blanks, standards, and quality control samples, i.e., undiluted plasma, plasma diluted 1:2 with PBS, brain homogenate diluted 1:4 in PBS, brain homogenate diluted 1:9 in aECF, brain homogenate diluted 1:19 in PBS. All samples were prepared by protein precipitation followed by dilution of the supernatant with an aqueous mobile phase. Quantification of the analyte in standards, quality controls, blanks and samples was performed using MassLynx 4.1/4.2 and TargetLynx (Waters, UK). Details on chromatographic and mass spectrometric conditions are presented in Supplementary Tables S2, S3. The runs were accepted when: (i) the calibration curve had a coefficient of determination (R^2^) of at least 0.99, regardless of the regression method; (ii) ≥ 75% of non-zero standards were within ± 15 % of their nominal values; (iii) all unknown samples were within the range of the standard curve; (iv) ≥ 67% of the quality control samples were within ± 15 % of their respective nominal values; (v) the calculated concentration in the single blank was less than the lower limit of quantification (which was set to the lowest standard level).

### 2.7 Data presentation and statistical data analysis

The extent of brain regional BBB and BSCB transport for selected drugs was assessed using K_p,uu,ROI_ calculated as :


(4) $$\begin{array}{l} {\text{K}}_{\text{p},\text{uu},\text{ROI}}=\frac{{\text{K}}_{\text{p},\text{ROI}}}{{\text{f}}_{\text{u},\text{plasma }}\;*\;{\text{V}}_{\text{u},\text{brain}}} \end{array}$$

Where K_p,ROI_ is the total CNS ROI-to-plasma concentration ratio determined at the end of 4-h intravenous infusion. K_p,uu,ROI_ values above unity indicate predominant active net uptake across the BBB and BSCB.

The extent of unbound drug transport across the BCSFB was calculated as the ratio between total CSF-to-plasma concentration ratio corrected for drug plasma protein binding. Drug CSF protein binding was considered negligible. K_p,uu,CSF_ values above unity indicate active net uptake across the BCSFB.

The ionization stage of the compounds is pH-dependent and driven by a physiological pH gradient between plasma (pH 7.4), ISF (pH 7.3), cytoplasm (pH ~ 7) and acidic subcellular compartments such as lysosomes (pH ~ 5). A three-compartment pH partitioning model of K_p,uu,cell,pred_, where “pred” stands for predicted values, was used to predict the unbound drug cell partitioning coefficient (Fridén et al., 2011). The model is based on the pKa of the compound, physiological volumes and the pH of the relevant compartments: interstitium, cytoplasm and lysosomes, according to Fridén et al. (2011):


(5) $$\begin{array}{l} {\text{K}}_{\text{p},\text{uu},\text{cell},\text{pred}}={\text{V}}_{\text{ISF}}+{\text{K}}_{\text{p},\text{uu},\text{cyto},\text{pred}}\cdot \left({\text{V}}_{\text{cyto}}+{\text{V}}_{\text{lyso}}\cdot {\text{K}}_{\text{p},\text{uu},\text{lyso},\text{pred}}\right) \end{array}$$

Where V_ISF_, V_cyto_, and V_lyso_ are the physiological volumes of the ISF (0.20 mL/g brain), cytosol (0.79 mL/g brain), and lysosomes (0.01 mL/g brain), respectively. The brain tissue density was assumed to be 1 g/mL. The ratios of cytosolic-to-extracellular unbound drug concentrations (K_p,uu,cyto,pred_) and lysosomic-to-cytosolic unbound drug concentrations (K_p,uu,lyso,pred_) for the bases were calculated as:


(6) $$\begin{array}{l} {\text{K}}_{\text{p},\text{uu},\text{cyto},\text{pred}}=\frac{10^{\text{pKa}-\text{p}{\text{H}}_{\text{cyto}}}+1}{10^{\text{pKa}-\text{p}{\text{H}}_{\text{ISF}}}\;+1} \end{array}$$

(7) $$\begin{array}{l} {\text{K}}_{\text{p},\text{uu},\text{lyso},\text{pred}}=\frac{10^{\text{pKa}-\text{p}{\text{H}}_{\text{lyso}}}+1}{10^{\text{pKa}-\text{p}{\text{H}}_{\text{cyto}}}+1} \end{array}$$

Where pH_cyto_ = 7.06, pH_ISF_ = 7.3 and pH_lyso_ = 5.18, as determined by Fridén et al. (2011).

Estimation of K_p,uu,cell,obs_, where “obs” stands for observed, was performed using the following equation proposed by Fridén et al. (2007):


(8) $$\begin{array}{l} {\text{K}}_{\text{p},\text{uu},\text{cell},\text{obs}}={\text{V}}_{\text{u},\text{brain}}\cdot {\text{f}}_{\text{u},\text{brain}} \end{array}$$

Discrepancy between predicted and observed K_p,uu,cell_ values indicate the presence of active processes occurring at the level of the brain parenchymal cellular membrane.

The standard deviation (SD) for K_p,uu,ROI_ as well as K_p,uu,cell_ was calculated following the law of propagation of error, since they were derived from three (Equation 4) or two (Equation 8) parameters with an uncertainty for each parameter (Loryan et al., 2017). The propagation of uncertainty was estimated for both the product and quotient of two variables, A and B, using the following equations. Propagation of uncertainty of K_p,uu,ROI_ was calculated according to the quotient rule. Let A and B be variables with their respective SD σ_A_ and σ_B_ and set:


(9) $$\begin{array}{l} f=\;\frac{A}{B} \end{array}$$

Propagation uncertainty for a quotient, i.e., the SD of f, was then calculated as follows:


(10) $$\begin{array}{l} \sigma_{f}\approx \;|f|\times \sqrt{{\left(\frac{\sigma_{A}}{A}\right)}^{2}+{\left(\frac{\sigma_{B}}{B}\right)}^{2}-2\frac{\sigma_{AB}}{AB}} \end{array}$$

As abovementioned, the covariance was calculated as σ_AB_ = rσ_A_σ_B_. Considering the innate correlation between the variables, |r| = 0.5 was assumed in all formulas. A negative correlation, i.e., r = −0.5, was present between V_u,tissue_ and f_u,plasma_, V_u,tissue_ and f_u,tissue_ while all other parameters were positively correlated, i.e., r = 0.5.

Propagation of uncertainty of K_p,uu,cell_ was calculated according to the product rule. Let A and B be variables with respective SD σ_A_ and σ_B_ and set:


(11) $$\begin{array}{l} f=\;A\cdot B \end{array}$$

Propagation uncertainty for a product, i.e., the SD of *f*, was then calculated as follows:


(12) $$\begin{array}{l} \sigma_{f}\approx \;|f|\times \sqrt{{\left(\frac{\sigma_{A}}{A}\right)}^{2}+{\left(\frac{\sigma_{B}}{B}\right)}^{2}+2\frac{\sigma_{AB}}{AB}} \end{array}$$

The covariance σ_AB_ was calculated with the correlation r as σ_AB_ = rσ_A_σ_B_.

Statistical analysis of data was performed using GraphPad Prism 9.4.0 for Windows (GraphPad Software, San Diego, CA, USA). Shapiro-Wilk test and Kolmogorov-Smirnov test were used to analyze the normality and lognormality of the data distribution. The differences between the mean values of f_u,ROI_, K_p,ROI_, K_p,uu,ROI_ and V_u,brain_ were assessed by ordinary one-way analysis of variance (ANOVA). The Brown-Forsythe Test was included in the f_u,ROI_ analysis to compare the equality of variances. In the case of exclusion of an invalid value, mixed effect modeling analysis was used. Dunnett's multiple comparison test was used to compare mean ROI values to the mean whole brain values and Turkey's multiple comparison test was used to compare every mean ROI with every other mean ROI. Data is presented as mean and SD.

## 3 Results

### 3.1 Mapping the extent of transport across the CNS barriers for selected H^+^/OC antiporter substrates

Mapping the transport of total and unbound drugs across the BBB and BSCB was performed using the parameters K_p,ROI_ and K_p,uu,ROI_. These represent the ratio of total or unbound drug concentrations in brain regions or the spinal cord to plasma concentrations at steady-state conditions, respectively. Despite some inter-individual variability for each drug, total plasma concentrations remained relatively stable during the 4-h infusion, indicating the achievement of steady-state (Supplementary Figure S1). Plasma protein binding assessments showed that the fraction unbound in plasma, f_u,plasma_, ranged from 0.33 for bupropion to 0.85 for oxycodone (Table 2). Similar plasma protein binding capacities were observed between bupropion, pyrilamine, and diphenhydramine, as well as between oxycodone and tramadol.

**Table 2:** Unbound fraction (f_u_) for the selected drugs in regions of interest (ROI) and plasma.

**Parameter**	**Drugs**					
	**Pyrilamine**	**Diphenhydramine**	**Bupropion**	**Tramadol**	**Oxycodone**	**Memantine**
f_u,ROI_						
Whole brain	0.093 ± 0.012	0.061 ± 0.003	0.209 ± 0.031	0.496 ± 0.128	0.430 ± 0.072	0.071 ± 0.014
Cerebellum	0.103 ± 0.021	0.061 ± 0.007	0.170 ± 0.028	0.560 ± 0.187	0.421 ± 0.037	0.076 ± 0.012
Frontal cortex	0.095 ± 0.010	0.059 ± 0.013	0.177 ± 0.023	0.513 ± 0.099	0.427 ± 0.065	0.071 ± 0.006
Parietal cortex	0.092 ± 0.005	0.067 ± 0.005	0.149 ± 0.018	0.421 ± 0.065	0.457 ± 0.040	0.071 ± 0.009
Striatum	0.106 ± 0.009	0.074 ± 0.006	0.145 ± 0.015	0.459 ± 0.032	0.450 ± 0.077	0.088 ± 0.006
Hippocampus	0.103 ± 0.013	0.064 ± 0.003	0.142 ± 0.004	0.462 ± 0.064	0.396 ± 0.065	0.078 ± 0.004
Spinal cord	0.075 ± 0.013	0.057 ± 0.004	0.096 ± 0.008	0.449 ± 0.068	0.427 ± 0.049	0.071 ± 0.014
f_u,plasma_	0.386 ± 0.093	0.389 ± 0.053	0.327 ± 0.068	0.827 ± 0.027	0.853 ± 0.036	0.543 ± 0.037

Data are expressed as mean ± standard deviation, N = 3–9, n = 1–3. No statistical differences were identified in the regional drug tissue binding.

**Figure 1:**
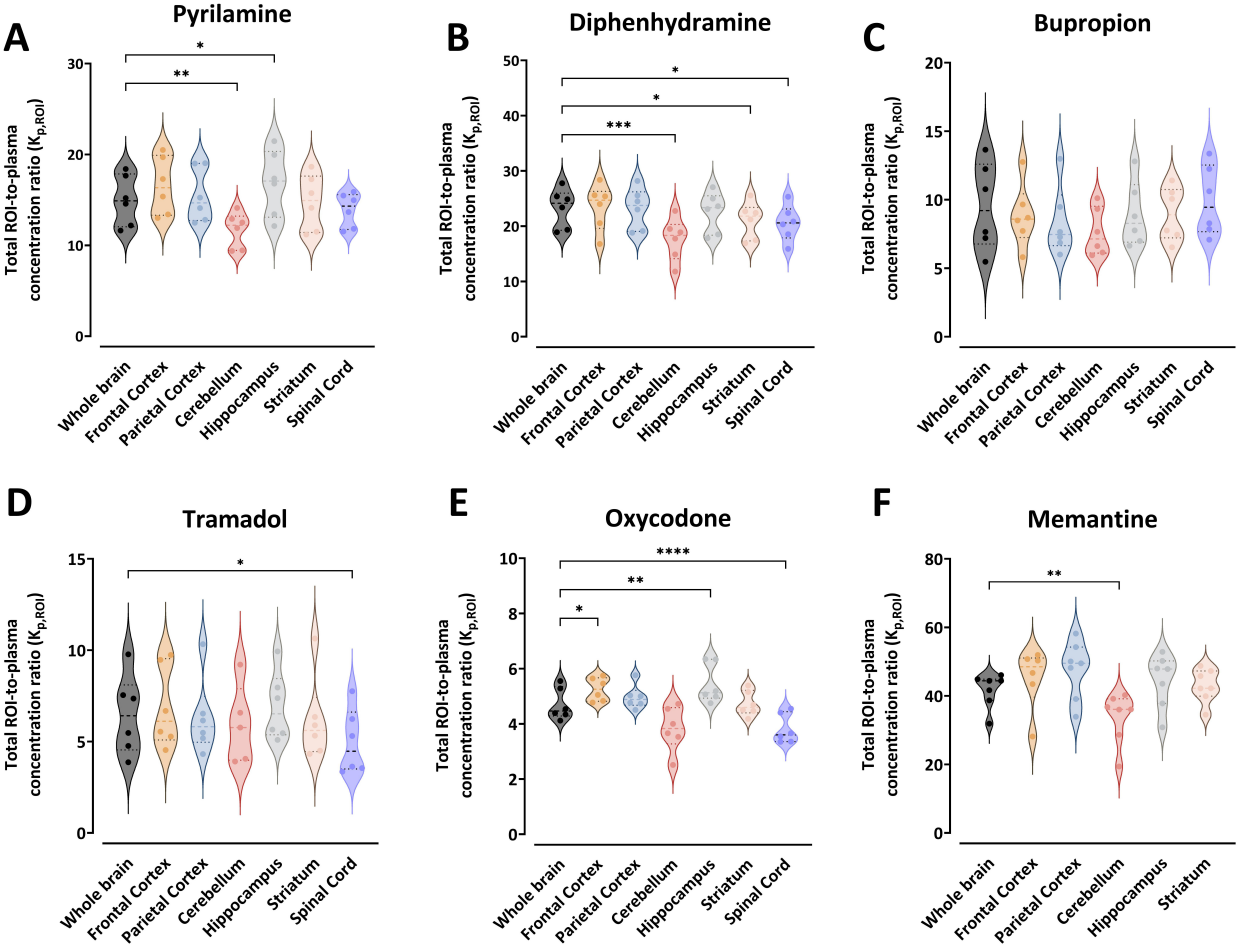
Violin plots representing the individual total region of interest (ROI)-to-plasma concentration ratio (K_p,ROI_) for pyrilamine **(A)**, diphenhydramine **(B)**, bupropion **(C)**, tramadol **(D)**, oxycodone **(E)**, and memantine **(F)** along with the median and quartiles. Comparisons of K_p,ROI_ values between the ROIs are performed using one-way ANOVA followed by *post-hoc* analysis using Dunnett's tests. Significant *p*-values for Dunnett's tests are presented ^*^p < 0.05, ^**^p < 0.01, ^***^p < 0.001, ^****^p ≤ 0.0001. NB. Differences on the Y-axes, and the absence of spinal cord samples for memantine.

The K_p,ROI_ values varied significantly between drugs. For example, the mean K_p,ROI_ in the whole brain ranged from 4.7 for oxycodone to 41.7 for memantine (Figure 1). The ranking of drugs by decreasing mean K_p,ROI_ was memantine > diphenhydramine > pyrilamine > bupropion > tramadol > oxycodone. There was minimal intra-region variability in K_p,ROI_, with a coefficient of variation not exceeding 22%. However, bupropion and tramadol showed higher unexplained intra-region variability, up to 38% observed in all regions (Figure 1). Generally, the lowest K_p,ROI_ values were observed in the cerebellum for all drugs. Compared to the cerebellum, K_p,ROI_ was significantly higher in the whole brain for pyrilamine (1.3-fold, *p* = 0.005), diphenhydramine (1.3-fold, *p* = 0.0003), and memantine (1.2-fold, *p* = 0.003).

**Figure 2:**
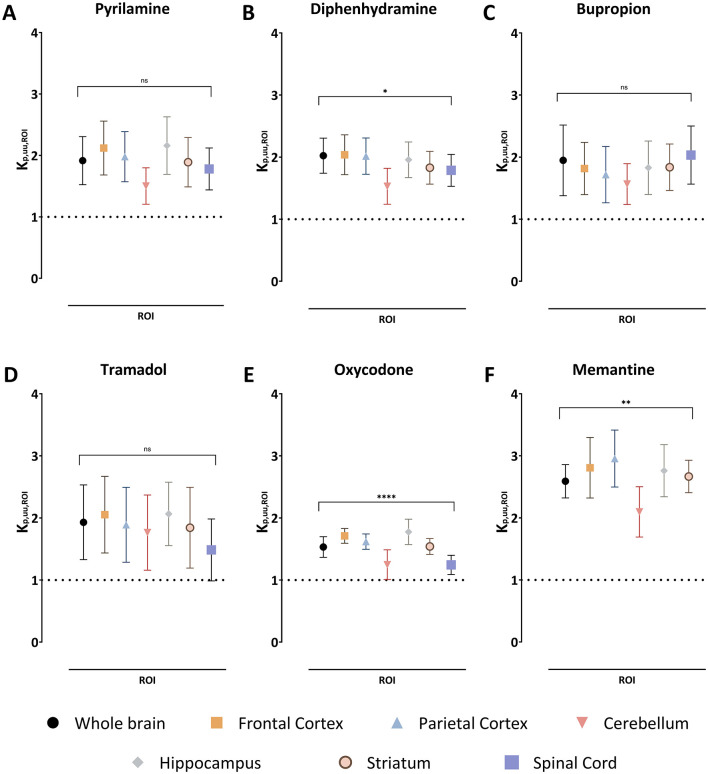
The extent of drug transport across the blood-brain barrier in the regions of interest (ROI) and blood-spinal cord barrier presented as a mean unbound ROI-to-plasma concentration ratio, K_p,uu,ROI_, together with the standard deviation for pyrilamine **(A)**, diphenhydramine **(B)**, bupropion **(C)**, tramadol **(D)**, oxycodone **(E)**, and memantine **(F)**. A K_p,uu,ROI_ of unity is indicated by a dashed line. Comparisons of K_p,uu,ROI_ values between the ROIs are performed using one-way ANOVA followed by post-hoc analysis using Dunnett's tests. Significant p-values for the ANOVA test at the group level are presented, *p < 0.05, **p < 0.01, ****p < 0.0001 and ns with *p* > 0.05. Data on post-hoc analysis are presented in the Results section. NB. The absence of spinal cord samples for memantine.

Since K_p,ROI_ is confounded by plasma protein binding and uptake and/or binding in CNS regions, K_p,uu,ROI_ was assessed to evaluate unbound drug transport across CNS barriers. K_p,uu,ROI_ indicated active net uptake for all organic cationic drugs studied across all regions, with low variability between drugs (Figure 2). For example, the mean K_p,uu,ROI_ in the whole brain ranged from 1.5 for oxycodone to 2.6 for memantine. The ranking by decreasing mean K_p,uu,ROI_ was memantine > diphenhydramine > bupropion = tramadol = pyrilamine > oxycodone (Figure 2, Table 3). Notably, there was no significant difference in BBB extent of transport among the investigated brain regions compared to the whole brain, though a trend toward lower K_p,uu,ROI_ in the cerebellum persisted (Figure 2). The estimated unbound brain ISF concentrations varied between the drugs, as a result of differences in binding properties in plasma and brain, and BBB transport. For instance, the mean C_u,brainISF,ss_ estimated in the whole brain was the highest for diphenhydramine and equal to 210 ng/g brain, while the lowest was detected for bupropion with 9.3 ng/g brain (Supplementary Table S4). Overall, the ranking of achieved steady-state C_u,brainISF_ was the following: diphenhydramine ≥memantine > oxycodone > tramadol> pyrilamine > bupropion. Additionally, a similar extent of active net uptake across the BCSB was observed for all drugs (Figure 2, Table 3).

**Table 3:** The extent of drug transport across CNS barriers.

**Drug**	**BBB**	**BSCB**	**BCSFB**
	**K**_p,uu,WB_	**K**_p,uu,SC_	**K**_p,uu,CSF_
Pyrilamine	1.92 ± 0.39	1.78 ± 0.34	0.68 ± 0.17^*^
Diphenhydramine	2.02 ± 0.28	1.79 ± 0.26	0.96 ± 0.13^*^
Bupropion	1.94 ± 0.57	2.03 ± 0.47	0.96 ± 0.27
Tramadol	1.93 ± 0.60	1.49 ± 0.49	0.86 ± 0.18^*^
Oxycodone	1.53 ± 0.17	1.24 ± 0.16	0.89 ± 0.07^*^
Memantine	2.60 ± 0.27^**^	NA	NA

Unbound partition coefficients across the blood-brain barrier in the whole brain (K_p,uu,WB_), blood-spinal cord barrier (K_p,uu,SC_), and blood-CSF barrier (K_p,uu,CSF_) for the drugs of interest presented as mean ± SD, n = 6 rats per drug, ^*^n = 5, ^**^ n = 7. NA, not available.

K_p,uu,CSF_ values in the *cisterna magna* ranged from 0.68 for pyrilamine to 0.96 for diphenhydramine and bupropion, suggesting predominant passive transport across the BCSFB. In general, drug transport across the BCSFB was lower compared to regional brain transport via the BBB, with a 1.6-fold difference for oxycodone and a 2.8-fold difference for pyrilamine when compared to mean K_p,uu,ROI_ in the whole brain (Table 3). This suggests that using CSF concentrations as a proxy for brain ISF concentrations may lead to an underestimation of brain ISF concentrations.

### 3.2 Intra-brain distribution for selected drugs with homogenous drug CNS tissue binding

The investigation of drug binding in brain regions and the spinal cord using equilibrium dialysis revealed that diphenhydramine had the highest binding properties, with a fraction unbound, f_u,brain_ of 0.061 in the whole brain (Table 2). In contrast, tramadol had the lowest binding capacity, with a f_u,brain_ of 0.496 in the whole brain (Table 2). Notably, there were no differences in binding capacity at the regional level for all studied drugs, confirming that the f_u,brain_ values measured in the whole brain can be applied to the individual regions.

**Figure 3:**
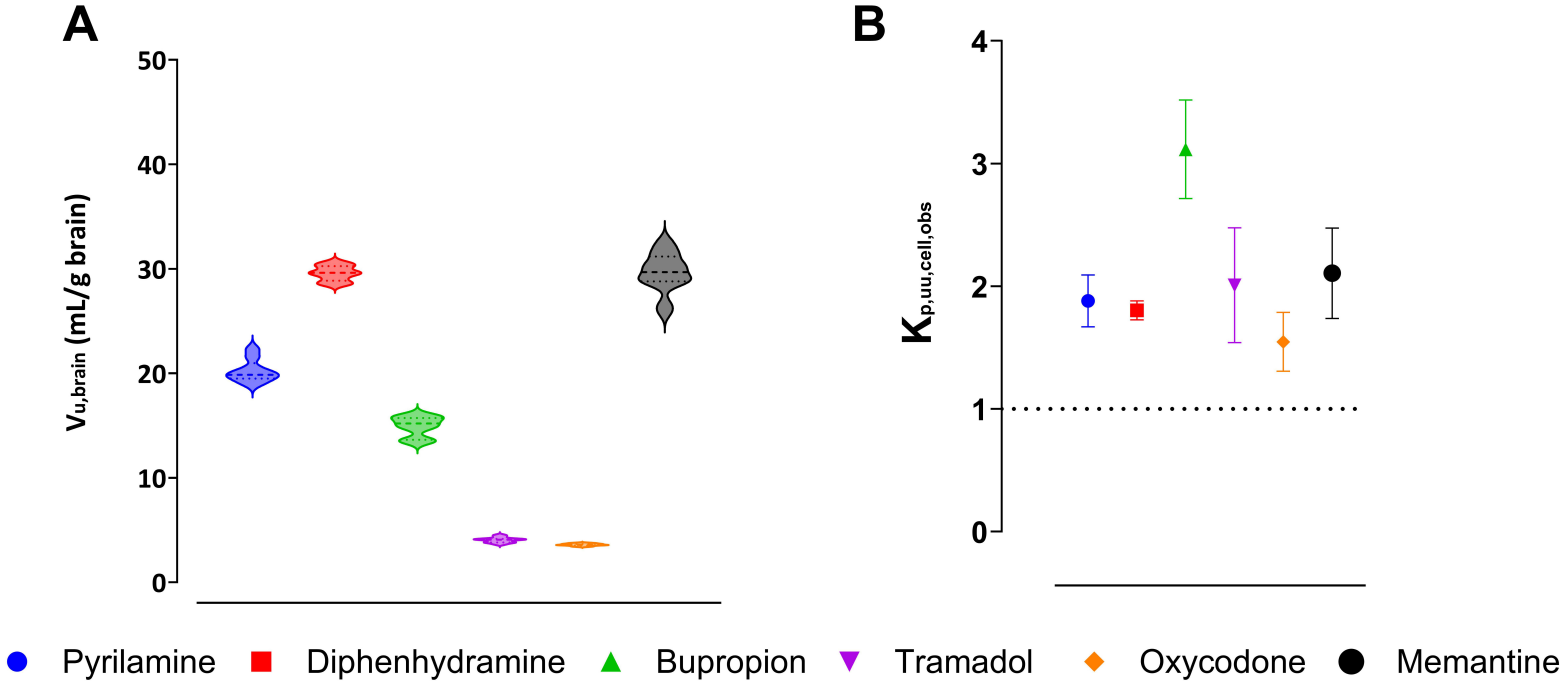
Intra-brain distribution of pyrilamine (blue), diphenhydramine (red), bupropion (green), tramadol (purple), oxycodone (orange), and memantine (black). Violin plots represent the individual unbound volume of distribution in the brain, V_u,brain_, with the median and quartiles indicated **(A)**. The extent of drug transport across the cellular barrier presented as the observed unbound brain intracellular-to-extracellular concentration ratio, K_p,uu,cell,obs_, with the mean and standard deviation indicated **(B)**. A K_p,uu,cell,obs_ of unity is indicated by a dashed line.

Assessment of binding and uptake in the brain slices, with preserved cellular integrity, showed that memantine had the highest unbound volume of distribution, V_u,brain_, of 29.7 mL per gram of brain tissue, while oxycodone had the lowest V_u,brain_ of 3.6 mL per gram of brain tissue (Figure 3A). The confirmation of region-independent f_u,ROI_ allowed us to use V_u,brain_ to estimate K_p,uu,ROI_ (Equation 4).

### 3.3 Extent of intracellular distribution for selected drugs

To estimate intracellular distribution of the investigated organic cations, the observed intracellular-to-unbound brain ISF concentration ratio, K_p,uu,cell,obs_, was calculated using Equation 8 (Figure 3B). The results showed that bupropion had the highest K_p,uu,cell,obs_ of 3.1, while oxycodone had the lowest value of 1.6. This indicates that all the drugs studied exhibited intracellular accumulation in brain parenchymal cells. Since all these drugs are weak bases with a high degree of ionization at physiological pH, the predicted intracellular-to-unbound concentration ratio, K_p,uu,cell,pred_, was estimated using the pH partitioning model (Fridén et al., 2011), which assumes passive diffusion of drugs across the cellular membrane. According to the model, all compounds had a predicted K_p,uu,cell,pred_ around 2.8 (Supplementary Table S5). Despite minor discrepancies between the observed and predicted K_p,uu,cell_ values for all drugs (except oxycodone), the results suggest that intracellular accumulation is likely governed by lysosomal trapping.

By using compounds capable of altering intracellular pH, such as the Na^+^/H^+^ ionophore monensin and the inhibitor of vacuolar-type H^+^-ATPase bafilomycin A1, we were able to modulate the intracellular distribution of drugs in the brain slice assay (Supplementary Figure S2). Preincubation with 50 nM monensin almost completely abolished intracellular accumulation, bringing K_p,uu,cell,obs_ close to unity. A similar but less pronounced effect was observed with the preincubation of brain slices with 10 nM bafilomycin A1. The results confirmed the role of pH partitioning in the intra-brain distribution of investigated drugs.

## 4 Discussion

This preclinical study represents the first systematic exploration of the CNS regional disposition of H^+^/OC antiporter substrates linked to DIS and MCAEs at therapeutically relevant plasma concentrations. Utilizing the CMA-ROI, we characterized the transport of unbound drugs across the BBB in specific CNS regions and the BSCB. Our results consistently showed a high level of active net uptake, with K_p,uu,ROI_ values exceeding unity across all assessed regions for pyrilamine, diphenhydramine, bupropion, tramadol, oxycodone, and memantine. Despite observing minor regional variations, there were no extensive differences when comparing the whole brain to specific regions of interest, indicating a region-independent active transport process. The transport across the BCSFB, evaluated at the *cisterna magna*, appeared to be primarily governed by passive transport or a balance of efflux and influx mechanisms. Additionally, we noted substantial intracellular accumulation, likely due to lysosomal trapping, for all studied drugs. These findings offer new insights into the CNS regional neuropharmacokinetics of these compounds, suggesting that while the drug uptake across the BBB is region-independent, active transport mechanisms can lead to high extracellular and intracellular drug concentrations in the CNS, which may contribute to neurotoxicity. This underscores the importance of comprehensive neuropharmacokinetic assessments and neurotoxicity profiling in the development of drugs that utilize this transport pathway.

The H^+^/OC antiporter, also known as the pyrilamine transporter or amine/H^+^ transporter, has been proposed to mediate the uptake of lipophilic alkaline drugs into the brain (Sadiq et al., 2011; Yamazaki et al., 1994; Okura et al., 2008; Sachkova et al., 2021). Various organic cation transporters, including organic cation transporters (OCTs), organic cation/carnitine transporters (OCTNs), and multidrug and toxin extrusion proteins (MATEs), have been identified, molecularly cloned, and characterized (Sweet, 2021). However, none of these transporters exhibits the same transport properties as the H^+^/OC antiporter (Doetsch et al., 2022; Sweet, 2021; Sachkova et al., 2022). The H^+^/OC antiporter operates as a secondary active transporter. Unlike primary active transport, which directly uses energy to move ions against their concentration gradients, secondary active transport leverages transmembrane electrochemical gradients to drive the uptake or efflux of nutrients, signaling molecules, drugs, and other ions across cell membranes. For an antiporter to function, it must bind to either a solute or ion(s) to facilitate transitions between outward-facing and inward-facing states, ensuring a stoichiometric exchange. Drew and Boudker have proposed that diffuse protein properties, such as accessible conformational ensembles, global and local dynamics, and allosteric networks, are crucial determinants of ion coupling under specific tissue conditions (Drew and Boudker, 2024). Concerning the H^+^/OC antiporter, several researchers suggest that it operates as a protein complex cycling through multiple conformational states to translocate its substrate molecules across the membrane (Sachkova et al., 2022; Kurosawa et al., 2022). Kurosawa et al., through knockdown screening in hCMEC/D3 cells, a model of the BBB, identified two proteins, TM7SF3 and LHFPL6, as molecular components of the H^+^/OC antiporter (Kurosawa et al., 2022). In a gain-of-function analysis, the authors used the HEK293 expression system to investigate the contributions of TM7SF3 and LHFPL6 to the uptake of H^+^/OC antiporter substrates including pyrilamine, tramadol and oxycodone. This discovery will advance the characterization of this transport system as a potential CNS drug delivery mechanism as well as promote the search of drugs that allosterically inhibit, activate, or alter the ion coupling of the H^+^/OC antiporter *in vitro* and *in vivo*.

From an *in vivo* perspective, it was essential to validate the previously reported active net uptake at the BBB, initially observed using brain microdialysis (Table 1), by employing the CMA-ROI method in this study. Although there was a slight discrepancy between the values obtained, all measurements consistently revealed a K_p,uu_ greater than unity, showing active uptake transport across the BBB and BSCB increasing the tissue concentrations, which may augment the risk for development of neurological side effects (Figure 2, Table 3). Notably, a higher-than-usual variability was observed for bupropion. This significant inter- and intra-subject variability, previously reported (Bhattacharya et al., 2023; Cremers et al., 2016), suggests potential concentration dependence. Therefore, a dose-dependent study including repeated dosing is necessary to further explore the variability in K_p,uu,ROI_ values, and to assess these differences also in overdose situations and after chronic administration. Our previous work using the CMA-ROI methodology highlighted distinct patterns in the extent of BBB transport across various brain regions and showed similar findings of lower K_p,ROI_ and K_p,uu,ROI_, with more efficient efflux in the cerebellum for marketed antipsychotics (Loryan et al., 2016). Significant regional differences in BBB transport were identified for P-gp substrates, with lower transport into the cerebellum compared to other regions, likely indicating higher P-gp activity in the cerebellum. High heterogeneity was observed for drugs like haloperidol, olanzapine, risperidone, and paliperidone, while quetiapine and clozapine showed minimal differences. Moreover, previous PET studies using the P-gp substrate verapamil, in both rats and humans, support the hypothesis of higher P-gp activity in the cerebellum (Müllauer et al., 2012; Kuntner et al., 2010; Bauer et al., 2010). These findings align with data showing higher P-gp protein expression in the dog cerebellum compared to the brain stem (Braun et al., 2017). Additionally, proteomic analyses of isolated brain microvessels from humans revealed higher expression of the junctional protein Claudin-5 in the cerebellum compared to the brain cortex (Chew et al., 2023), suggesting a tighter BBB in the cerebellum, which may further contribute to the lower drug exposure. These findings underscore the drug-specific nature of BBB transport evaluation. It is important to note that while the CMA-ROI method is effective for studying drug distribution in larger brain regions, it has limitations in resolving smaller areas. This limitation with spatial resolution has been addressed by a novel method, qMSI-uD, which enables the investigation of small brain regions and sub-regions using mass spectrometry imaging (Luptáková et al., 2021). Interestingly, the application of the qMSI-uD method to clozapine revealed significant regional differences, with the piriform cortex and lateral septum exhibiting up to a two-fold higher K_p,uu,ROI_ compared to the whole brain. Hence, refinement of the K_p,uu,ROI_ method could reveal potential differences in smaller regions that were not found when studying larger regions as revealed in the present study.

Similar to the extent of BBB transport, no differences were found in CNS tissue binding of the drugs studied, across the investigated regions of the brain and spinal cord. It suggests that similar properties govern drug binding throughout these areas. A similar trend was reported earlier (Gustafsson et al., 2019). The intra-brain distribution revealed intracellular accumulation, with all drugs showing a K_p,uu,cell_ greater than unity, indicating higher unbound drug concentrations in the intracellular compartment of the brain parenchyma, than in the interstitial fluid (Figure 3, Supplementary Table S5). This observation aligns with predictions based on the pH partitioning model (Fridén et al., 2011).

The hypothesis that the H^+^/OC antiporter plays an endogenous role in regulating brain extracellular and, possibly, intracellular pH led us to study the relationship between intracellular pH changes and drug uptake into the brain parenchymal cells. Lysosomal pH modulators, such as monensin and bafilomycin A1, can alter the transport of hydrogen ions within cellular compartments, increasing pH and reducing the trapping of weak base compounds in lysosomes. Monensin acts as a Na^+^/H^+^ ionophore, forming a complex with sodium ions and facilitating their transport across the membrane while exchanging protons (Fridén et al., 2011; Siebert et al., 2004; Lake et al., 1987). Once the sodium ion is released, monensin is re-protonated and can cross the membrane again. Bafilomycin A1, a macrolide, inhibits the vacuolar-type H^+^-ATPase, a proton pump that acidifies intracellular organelles (Cotter et al., 2015; Kane, 2021; DrugBank, 2020). Weak basic drugs are often trapped in lysosomes due to the acidic environment, which alters the drug molecules' charge and prevents them from diffusing through membranes. The similarity in functional representation between pH partitioning and active uptake makes dissection of both processes challenging, as in this case both are sensitive to pH changes. Pre-incubation of brain slices with monensin and bafilomycin A1 reduced the K_p,uu,cell_, with monensin bringing it close to unity, suggesting that observed brain intracellular accumulation is governed by pH partitioning. However, recent studies have shown that the lysosomal membrane-localized transporter SLC49A4 is involved in the lysosomal export of pyrilamine (Akino et al., 2023). Functional analyses using a plasma membrane-localized SLC49A4-AA mutant demonstrated H^+^-coupled transport for pyrilamine, and various H^+^/OC substrates were found to inhibit pyrilamine transport by SLC49A4-AA, indicating they may be competing substrates. These findings suggest that drug transport across the cellular and lysosomal membrane may involve not only passive transport but also an active transport component. Our research indicates that intracellular pH variations can significantly impact drug transport and may have implications for neurotoxicity. Systematic investigation is warranted to understand these potential effects under different conditions.

An important contribution from this study is the provision of detailed *in vivo* CNS exposure data, which can be leveraged in the design of *in vitro* studies where compound-specific therapeutically relevant brain ISF concentrations provided here could be used. Previous research has utilized these drugs to explore various neurotoxicological mechanisms, including *in vitro* electrophysiology assays (Zhai et al., 2023), transcriptomics signature fingerprints (Lipponen et al., 2023) and RNA editing of receptors, such as the serotonin 2C receptor (Cavarec et al., 2013). These studies aim to develop *in vitro* models capable of predicting neurotoxicity. While these *in vitro* studies offer valuable insights into the potential mechanisms underlying DIS and MCAEs, they often employ drug concentrations in the micromolar range, which far exceed the concentrations achievable *in vivo* in brain extracellular fluid at therapeutic doses. This huge discrepancy in drug concentration is a key factor that can lead to differences between *in vivo* and *in vitro* toxicity findings. Addressing this gap is crucial for improving the accuracy of *in vitro* models in predicting clinical neurotoxicity.

## 5 Conclusions

Variability in the extent of transport across CNS barriers and cellular barriers measured by K_p,uu,ROI_ and/or K_p,uu,cell_, resulting in differences of unbound drug exposure in the brain extracellular and intracellular fluids, is likely a significant factor contributing to the neurotoxic effects of drugs that are otherwise considered safe. In this preclinical study, we confirmed that the investigated substrates of the proton-coupled organic cation H^+^/OC antiporter exhibit active net uptake across both regional blood-brain and cellular barriers at therapeutically relevant plasma concentrations, leading to substantial brain extracellular and intracellular exposure. The intra-brain distribution of these drugs was found to be highly sensitive to changes in intracellular pH, which are often associated with various brain pathologies. A more detailed characterization of the brain disposition of investigated drugs under pathological conditions using animal disease models, with translational mathematical modeling supporting studies, may help identify patient populations or groups vulnerable to drug-induced seizures and mood- and cognitive-related adverse events.

## Data Availability

The raw data supporting the conclusions of this article will be made available by the authors, without undue reservation.
