# Supplementary material for: Region-independent active CNS net uptake of marketed H+/OC antiporter system substrates
Source: Front Cell Neurosci. 2024 Oct 29;18:1493644. doi: 10.3389/fncel.2024.1493644 (PMC11554538; doi:10.3389/fncel.2024.1493644)
Supplement: Supplementary file 1 [file Data_Sheet_1.pdf]

Supplementary Material

1 Supplementary Data

Supplementary materials include two figures (S1, S2) and four tables (S1-S4).

2 Supplementary Figures and Tables

2.1 Supplementary Figures

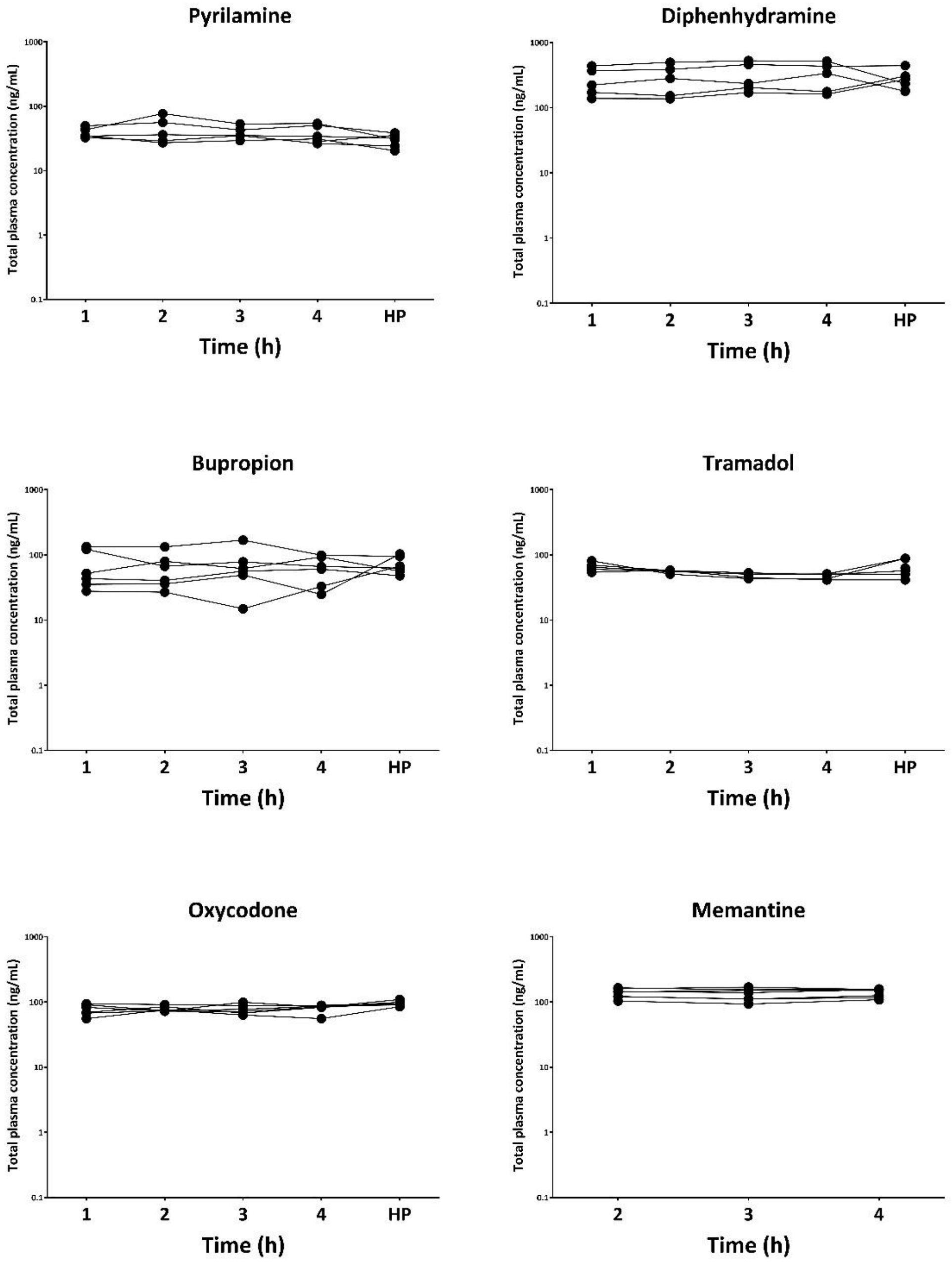

Supplementary Figure 1. Total plasma concentration-time profiles of pyrilamine, diphenhydramine, bupropion, tramadol, oxycodone, and memantine, confirm the achievement of a steady-state. HP refers to a terminal heart puncture sample. NB. For memantine there are only three sampling time points at 2, 3, and 4 hours, confirming the achievement of a steady-state in plasma.

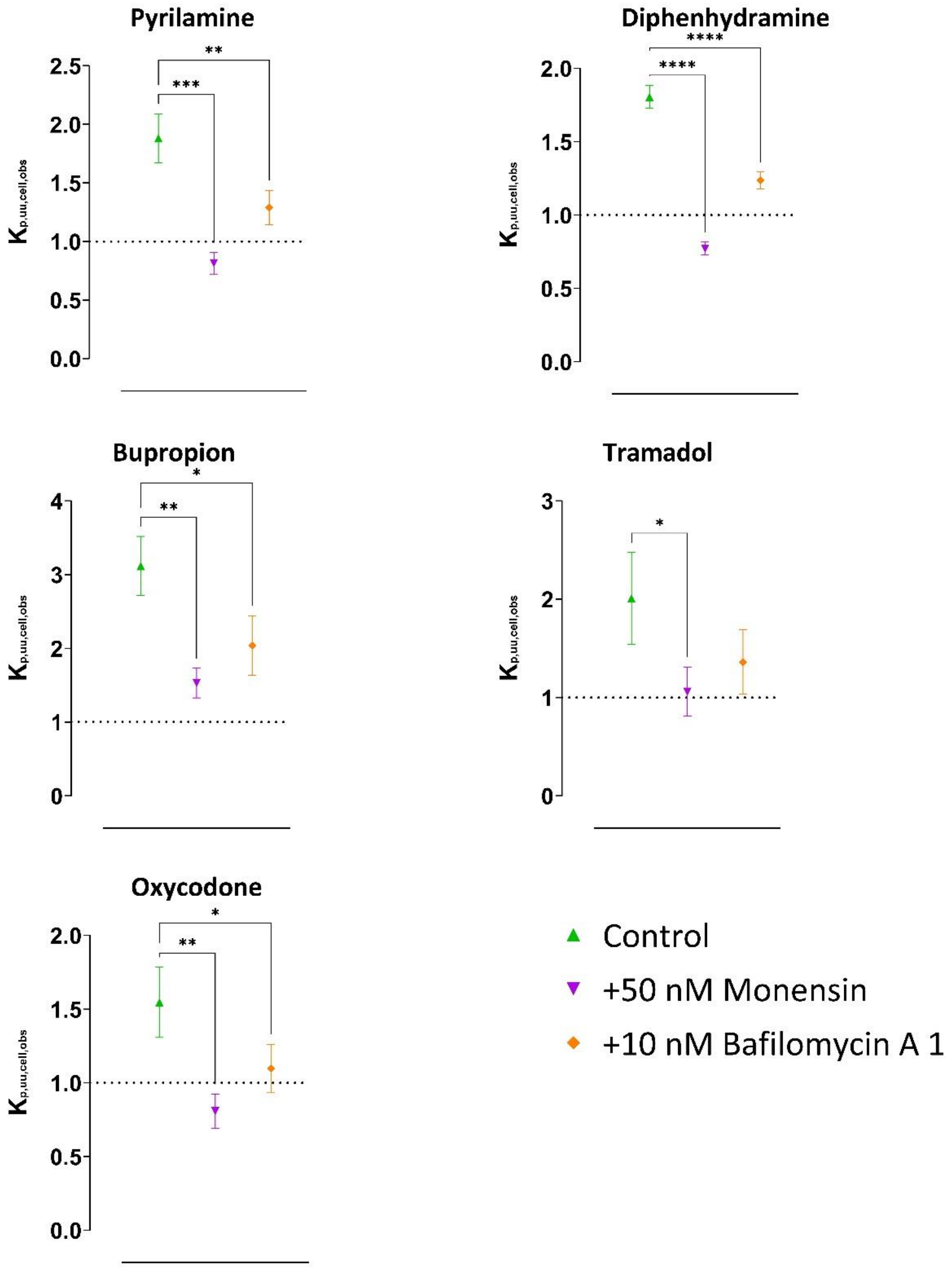

Supplementary Figure 2. The role of pH modulation on the intracellular distribution of organic cations. The extent of drug transport across the cellular barrier presented as a mean of observed unbound brain intracellular-to-extracellular concentration ratio, $K_{p,uu,cell,obs}$, together with the standard deviation. Three conditions have been investigated: control, preincubation with 50 nM of monensin, preincubation with 10 nM bafilomycin A1. $K_{p,uu,cell,obs}$ obtained during the pH modulated conditions were compared with the control using ordinary one-way ANOVA and Dunnett's multiple comparisons test for each compound. *p≤0.05, **p≤0.01, ***p≤0.001, ****p≤0.0001. $K_{p,uu,cell,obs}$ of unity is indicated by a dashed line.

2.2 Supplementary Tables

Table S1. Dosing regimen (period, intravenous infusion rate), drug concentration in the infusion solution and vehicles for the selected drugs in the pharmacokinetic studies in rats.

**Drug**	**Dosing regimen**				**Vehicle**	**Targeted steady-state total plasma concentration (ng/mL)**
	Loading dose		Maintenance dose			
	Period (h)	Rate (mg/h/kg)	Period (h)	Rate (mg/h/kg)		
**Pyrilamine**	0-0.17	3.0	0.17-4	0.22	Saline	65
**Diphenhydramine**	0-0.17	9.7	0.17-4	2.8	Saline	300
**Bupropion**	0-0.17	4.3	0.17-4	0.62	Saline	50
**Tramadol**	0-0.17	3.8	0.17-4	0.37	Saline	100
**Oxycodone**	N.A.	N.A.	0-4	0.3	Saline	60
**Memantine**	0-0.17	19.6	0.17-4	1.9	10% HPbCD[§] in saline	100

[§]HPbCD - 2-hydroxypropyl-β-cyclodextrin

Table S2. Liquid chromatography conditions used for bioanalysis of selected drugs.

Drug	Time (min)	Flow rate (mL/min)	Percentage of mobile phase A (%)	Percentage of mobile phase B (%)	Retention time
**Pyrilamine** § **Diphenhydramine** § **Bupropion** § **Tramadol** §	Initial	0.3	95.0	5.0	2.07 2.16 2.10 1.80
	1.00	0.3	95.0	5.0	
	2.00	0.3	5.0	95.0	
	3.00	0.3	5.0	95.0	
	3.20	0.3	95.0	5.0	
	Initial	0.3	95.0	5.0	
	1.00	0.3	95.0	5.0	
	2.50	0.3	55.0	45.0	
**Oxycodone** §	3.50	0.3	55.0	45.0	2.27
	4.00	0.3	10.0	90.0	
	4.50	0.3	95.0	5.0	
	4.80	0.3	95.0	5.0	
**Memantine**#	Initial	0.8	90.0	10.0	2.75
	4.00	0.8	5.0	95.0	
	4.50	0.8	5.0	95.0	
	5.00	0.8	90.0	10.0	

Mobile phase A consisted of 0.1% FA (formic acid) in MilliQ water, and mobile phase B consisted of 0.1% FA in acetonitrile. §AQUITY UPLC BEH C18 column, 50 mm × 2.1 mm, 1.7 µm, was used. #HyPURITY C18 column, 50×4.6 mm, 3µm, from Thermo Scientific, USA was used.

Table S3. Detailed mass spectrometric conditions for the selected drugs.

Analyte	Internal standard (IS)	Capillary voltage (kV)	Cone voltage (V)	Collision energy (V)	MRM transition	
					Analyte	IS
**Pyrilamine**	NA	0.5	30	12	286.20→ 121.00	NA
**Diphenhydramine**	Diphenhydramine-D5	0.6	30	12	256.20 → 167.10	259.20 → 167.10
**Bupropion**	Bupropion-D9	0.5	30	12	240.10 → 184.10	249.20 → 185.10
**Tramadol**	Tramadol-^{13}CD3	0.6	30	15	264.20 → 58.00	268.20 → 58.00
**Oxycodone**	Oxycodone-D6	0.5	10	19	316.1→ 298.1	322.2 → 304.1
**Memantine**	Memantine -D6	3	40	14	180.10 →162.90	186.00 → 168.90

The MS/MS was set to a positive electrospray mode for ionization. Nitrogen gas was used as desolvation gas and the flow rate was set to 1000 L/h with a temperature of 600°C (only for memantine: 450°C). The source temperature was set to 150 °C (only for memantine: 125°C).

Table S4. Unbound steady-state drug concentrations in brain interstitial fluid (ISF, $C_{u,brainISF,ss}$) and cerebrospinal fluid (CSF, $C_{tot,CSF,ss}$) for the selected drugs in the pharmacokinetic studies in rats.

Drug	$C_{u,brainISF,ss}$	$C_{tot,CSF,ss}{}^{\$}$
**Pyrilamine**	21.6±2.0	7.7±1.9
**Diphenhydramine**	210.8±84.8	94.9±32.3
**Bupropion**	9.3±4.6	21.9±5.6
**Tramadol**	95.0±9.4	47.7±8.2
**Oxycodone**	124.0±16.7	72.4±10.6
**Memantine**	194.3±38.5	NA

$Total CSF concentrations at steady-state are assumed to be equal to the unbound due to neglection of drug binding in CSF. NA=not available

Table S5. Observed and predicted, based on the pH portioning model, intracellular distribution parameters. See the explanation in the text.

**Drug**	**pKa**	$K_{p,uu,cyto}$	$K_{p,uu,lyso}$	$K_{p,uu,cell,pred}$	$K_{p,uu,cell,obs}$
**Pyrilamine**	8.76	1.713	74.393	2.828	1.88
**Diphenhydramine**	9.30	1.730	75.429	2.872	1.804
**Bupropion**	8.60	1.703	73.759	2.801	3.116
**Tramadol**	9.7	1.735	75.687	2.884	2.008
**Oxycodone**	9.1	1.726	75.181	2.862	1.547
**Memantine**	10.70	1.738	75.841	2.890	2.106
